# Development and evaluation of a tool (named Evidence Brief) to communicate allied health research translation

**DOI:** 10.1186/s12913-025-13421-1

**Published:** 2025-10-21

**Authors:** Tilley Pain, Gail Kingston, Stephen Perks, Amy Brown, Lisa Thompson

**Affiliations:** 1 Allied Health Governance, Townsville Hospital and Health Service, Townsville, Qld Australia; 2 College of Public Health, Medical and Veterinary Science, James Cook University, Townsville, Qld Australia; 3 Townsville Hospital Pharmacy, Townsville Hospital and Health Service, Townsville, Qld Australia; 4 Townsville Cancer Centre, Townsville Hospital and Health Service, Townsville, Qld Australia; 5 College of Medicine and Dentistry, James Cook University, Townsville, Qld Australia; 6 Townsville Institute of Health Research and Innovation, Townsville Hospital and Health Service, Townsville, Qld Australia

## Abstract

**Background:**

An allied health research capacity building initiative at a regional Australian public health service has increased research activity significantly. To demonstrate the value of allied health research activity a short one-page narrative was developed to communicate the impact of allied health research. This paper evaluates the use of the narrative at this healthcare organisation.

**Methods:**

A multiple case study design was used for the evaluation. Three cases written as narratives were chosen and one comparison case without a narrative. The cases were investigated via organisational document review, policies, or guidelines, and published journal articles, plus semi-structured interviews were conducted with relevant stakeholders. Analysis was conducted in four stages: case context and description, within-case analysis, cross-case analysis and interpretation and evaluation using thematic analysis.

**Results:**

Document analysis revealed the rationale and evidence for the practice change, the instigator of change and enablers. Cross-case analysis identified commonalities such as an expanded scope of practice, clinician-led change, and the inclusion of salient stakeholders to ensure that translation occurred. Differences included the timing of funding and the reach of change.

**Conclusion:**

The one-page narrative (named Evidence Brief) effectively describes a change in clinical practice because of allied health research or quality improvement projects. Evidence Briefs have potential to act as a research measure with each Evidence Brief acting as a unit of change so that over time, accumulated Evidence Briefs may be used to measure clinical practice change resulting from research. Future research by this team will obtain feedback from management to determine the value of Evidence Briefs as a communication tool and to evaluate the extended use of them across other disciplines and healthcare organisations.

Keywords: case study, evaluation, communication, clinical practice change, research impact

**Supplementary Information:**

The online version contains supplementary material available at 10.1186/s12913-025-13421-1.

## Introduction

Allied health professionals within health care organisations are becoming increasingly research active [1–3]. The benefits of increased allied health research activity include efficiencies derived from expanded scope roles [4], improved access via substitution models [5, 6] and reduced time to translation as a result of clinician-led research conducted in healthcare organisations enabling shorter translation time [7] and reducing research waste [8]. More broadly, research active healthcare organisations are associated with patient, staff and organisational benefits [9] and improvements in healthcare performance [10]. To justify investment in research, it is important to communicate the benefits of research to management within healthcare organisations or to funding institutions in a format they can understand. One communication mechanism is the impact case study. However, communicating the benefit of research through impact case studies has been shown to be inconsistent, requiring more systematic reporting of translation pathways and a need for greater transparency to estimate costs and benefits of the research [11]. Despite these challenges, case studies are used to provide a plain language summary of research impact [12]. Allied health research at the Townsville and Hospital and Health Service (THHS) have developed a one-page narrative to describe clinical practice change from research or quality improvement activity. The narrative is called Evidence Brief.

## Background

The Evidence Brief was developed by the allied health Research Fellow at THHS to report the clinical impact of the role. The Research Fellow position was funded in 2010 to build research capacity of allied health professionals as part of a state-wide initiative [13]. In the tight fiscal environment of Queensland Health at that time, the Research Fellow was required to justify investment in a non-clinical role. A scan of research reporting tools found the Deeble Institute’s Health Policy Briefs which included Issues Briefs, Evidence Briefs and Perspective Briefs [14]. The format of the Evidence Brief consisted of three headings (1) Policy issue, (2) what does the evidence say and (3) what does this mean for policy makers [15]. The Research Fellow contacted the Australian Health and Hospital Association (the host organisation for the Deeble Institute) and asked permission to modify and use the concept for reporting the clinical impact of research performed by allied health at THHS. Permission was provided and modifications included simplification of the headings to (1) Issue, (2) Evidence and (3) Practice change.

The Evidence Brief was used as a communication tool in the Health and Wellbeing unit (under which most Allied Health disciplines reported at the time) of THHS from 2014. The Research Fellow summarised the outcome of local allied health research using the three-point format of the Evidence Brief ensuring it was kept to a single page. The Evidence Brief was submitted to management via the Director of Allied Health as part of the organisational reporting process. After several years of its use, a small collection gathered suggesting that in an aggregated format they could be used to indicate allied health research impact on clinical practice. This study evaluated the Evidence Brief’s effectiveness as a communication tool and its potential to be used as a measure of the clinical impact of allied health research at THHS.

## Method

### Design

A multiple case study design was used to explore the complex dynamic of translating research into practice in a clinical setting [16, 17]. Three cases were purposively selected from completed allied health research projects and one quality improvement (QI) project. The purposive selection aimed for variation across disciplines, research methods, and workforce levels. The quality improvement project was purposively included to test its appropriateness for reporting in this format as many Allied Health projects are quality improvement, known to impact clinical care, but not captured in traditional research metrics. The theoretical framing for the study drew on the Donabedian model as a conceptual model or framework to help examine the quality of care using information about structures, process and outcomes [18]. The study is reported using the consensus standards for organisational case studies (Supplement 1).

### Aim

The aim of this study was to explore whether the practice change communicated by the Evidence Brief had occurred within the clinical environment and if the change in practice continued since its introduction to determine its effectiveness as a communication tool. In addition, the evaluation of selected Evidence Briefs tested its potential to act as a measure of impact on clinical practice.

### Setting

This research was conducted at Townsville Hospital and Health Service (THHS). THHS is a regional publicly funded health service in regional northern Queensland serving a catchment of 148,000 square kilometres with approximately 700,000 people. The health service includes a tertiary referral hospital in a large regional city and nine rural health facilities serving communities categorised on the Modified Monash Model (MM) based on remoteness and population size from category 2 (regional city) through to 7 (very remote) [19]. The clinical workforce at THHS is comprised of four main streams: medicine, nursing and midwifery, dental and allied health.

Research is a strategic pillar of THHS with the establishment of the Townsville Institute of Health Research and Innovation and officially opened at THHS in 2018. The Institute provides administrative support to the Human Research Ethics Committee (HREC), site authorisation via Research Governance Office and research support for clinicians in the areas of epidemiology, statistics and digital solutions [20]. Almost 1.5 million Australian dollars (AUD) of internal funding was disbursed by the Institute to clinician researchers in 2022 and clinicians produced 274 peer reviewed publications [21]. Thirteen teams have formed research groups within THHS including an allied health research team.

### Context

Allied health professionals are research active at THHS. Allied health clinician researchers are supported by two fractional Research Fellows employed under a state-wide initiative to build allied health research capacity [13]. Since 2010, the allied health workforce at THHS has increased their research experience in consuming and producing research with higher levels of activity in the consuming research via research translation or evidence-based practice [22]. Despite the high level of research support at the organisational level, allied health research is less productive than the other clinical streams. Of the AUD1.5 million in funds disbursed in 2022, allied health received approximately AUD200,000 and produced 23 of the 274 peer reviewed papers [21]. Allied health professionals at THHS are more experienced at consuming research (via evidence-based practice or translation) than producing research in the form of published papers. Therefore, a way to communicate allied health research (both consuming and producing) and demonstrate its value to organisational management was needed in a mechanism other than the traditional metrics of publications and grant success, leading to the Evidence Brief development and implementation.

### Data collection

Data were collected by document review and semi-structured interviews. The documents included completed Evidence Briefs, organisational workplace instructions, internal project reports and peer-reviewed papers. The interviews were conducted face to face or by telephone with allied health clinicians who conducted the projects, and managers and clinicians in departments in which a practice change occurred. The interviews followed a guide specifically developed for this study (Supplement 2). The interviews sought information regarding the stakeholders involved in introducing the change, the sustainability of change and the documents or processes associated with the change.

Participants were sent an email inviting them to participate in the study and explaining the purpose of the study and intention to publish findings. All invited participants consented to be interviewed via return email prior to interview.

### Data analysis

For this evaluation, investigators first read the Evidence Brief associated with the research projects and the Final Report of the quality improvement project. These documents were read to obtain an overview of each case and additional details were sought by reviewing associated documents and interviews with relevant stakeholders. Specific case study data included the date of ethics approval, time from approval to publication, number of citations, and when the study was introduced into practice.

**Fig. 1:**
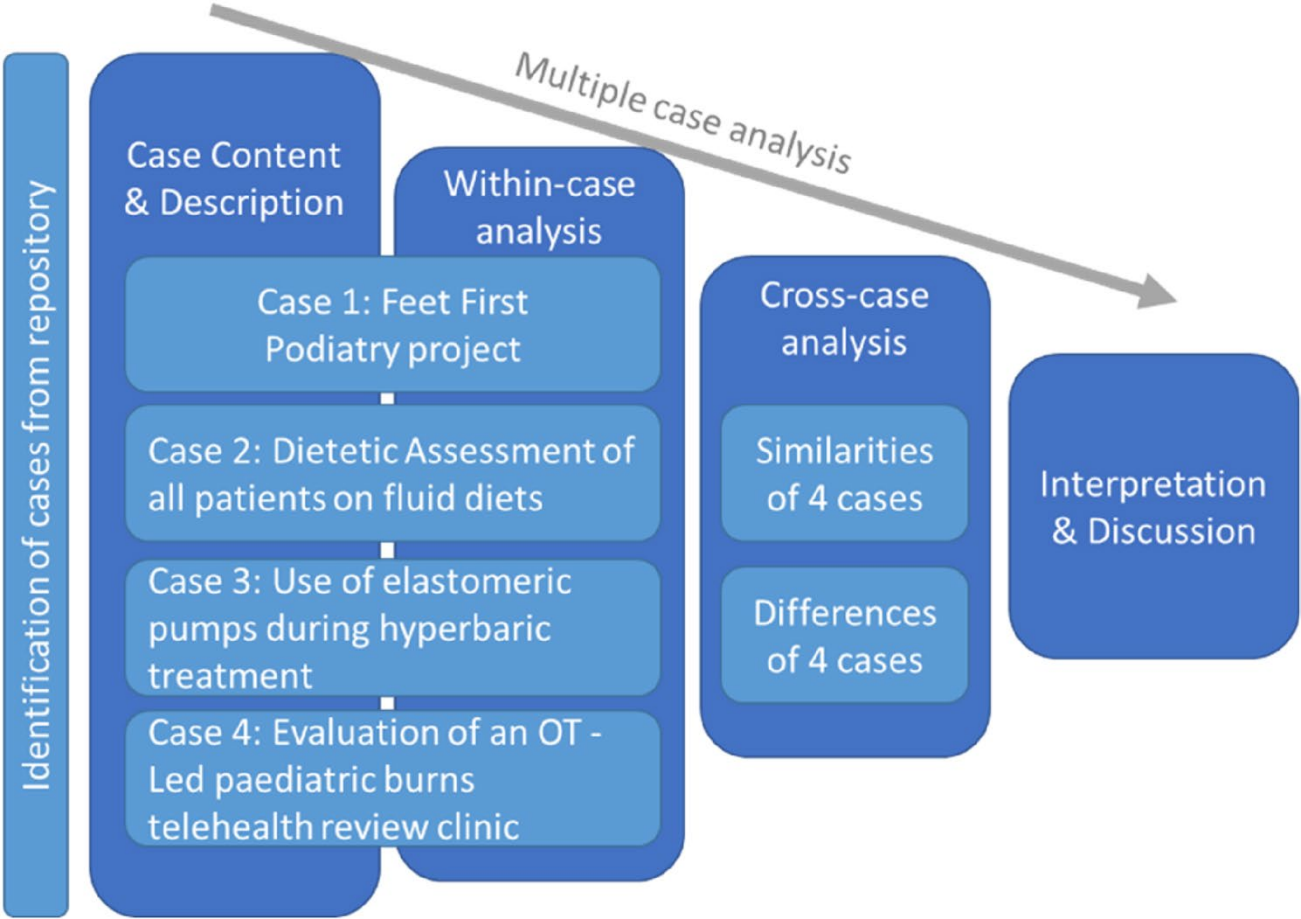
Modified template for coding a case study using a multiple case approach. OT = Occupational therapy

A template for coding case studies directed the initial description of each case and its context (Fig. 1). A within-case analysis followed, highlighting codes and categories relevant to the change in clinical practice and similarities between the cases were highlighted in a cross-case analysis. The final phase of analysis provided interpretations across the cases and reflections on whether the EB suitably captured elements of practice change (e.g., stakeholder involvement, clinical instructions or protocols) to enable the use of the EB as a measure of translation. All authors independently performed the analysis and then met to discuss the findings.

## Results

### Context and case description

#### Case 1 Dietetic intervention for patients on fluid diets (Attachment 1)

A group of THHS dietitians, who were novice researchers, designed and conducted their first research study to build research capacity and capability within the department. They were supported by the THHS allied health Research Fellow and an academic from James Cook University. The team obtained ethics approval from the THHS Human Research Ethics Committee in 2014 and received no funding. The study took over two and a half years for the dietitians to complete in their own time. The study was an observational study of 57 patients on fluid diets. The nutrient intake of patients was observed and recorded for comparison between those receiving dietetic intervention and those who did not. The study was published in a peer reviewed journal with four citations to date, and a procedure for patients on fluid diets to be referred to a dietitian if they were on a fluid diet for three days or longer was written [23]. The dietitians liaised with nursing colleagues on the wards to implement the practice change in February 2016.

#### Case 2 Use of Baxter pumps in the hyperbaric chamber (Attachment 2)

A senior pharmacist whose PhD topic was the stability of antibiotics in tropical conditions designed and conducted the study. The project received AUD$6,245 from Baxter International and was approved by the THHS Human Research Ethics Committee in 2015. The candidate was supervised by the THHS Research Fellow and an academic from James Cook University. The study questioned the ability to use Baxter Elastomeric pumps for patients with indications for continuous antibiotic infusion and concurrent hyperbaric treatment. Prior to the study, usual practice was for the pumps to be removed during hyperbaric treatment meaning these patients receive a reduced dose of antibiotics during that 24-hour period. By imitating the conditions for optimal pump use on healthy volunteers, flow rate of the pump was measured during hyperbaric pressures. No difference was observed between normo-baric and hyperbaric flow rate from the Baxter pump. All the Hyperbaric Medicine team were authors on the paper which has received six citations, and the practice change was introduced on consensus of all members of the team in March 2017 [24]. 

#### Case 3 Occupational therapy-led paediatric burns rehabilitation (Attachment 3)

The study was designed and conducted by three hospital occupational therapists one of whom had a PhD. Additional research support was provided by the THHS Research Fellow. The project was funded by Allied Health Professions Office of Queensland for innovative Models of Care (AUD$51,240) and THHS Study Education Research Trust Authority (AUD$47,153) with ethics approval granted by the THHS Human Research Ethics Committee in 2017. The new model of care addressed the lack of follow up rehabilitation for rural and remote children who had sustained severe burn injuries. The Project included developing clinical and administrative guidelines for occupational therapist-led rehabilitation via telehealth. An evaluation was conducted to determine the change in follow up support for rural and remote children compared to metropolitan counterparts. The occupational therapist-led service was implemented using program logic and included paediatric surgeons and nursing staff as stakeholders. The study was published in a peer reviewed journal [25] and has 7 citations.

#### Case 4 Feet first podiatry project

THHS received funding to expand ambulatory high risk foot services (AUD$66,639). Prior to expending the funds, the podiatry team discussed the best use of funds was to develop a model of care to overcome the high foot ulcer activity in a region with a small podiatry workforce. A podiatrist was funded to develop the clinical guidelines for rural and remote allied health assistants or Indigenous Health Workers to assess community members with high-risk feet. The project included a foot referral pathway and a toolkit of resources for the rural and remote sites. The service was registered as a quality improvement project and followed a logic framework for introducing new programs. The project produced a written report for management and has been presented at a state-wide forum.

### Within-case analysis

Analysis of the document review and stakeholder interviews from each case resulted in six themes. Themes included the practice change, rationale for the change, origin of the evidence, key steps in implementing change, the instigator of change and whether it was ongoing. The rationale for change in each case was driven by a patient level need or gap in service provision.

*The lack of consistent and coordinated Podiatric services in these areas [rural and remote] creates a highly reactive and ‘crisis-led’ approach to care for patients at risk of a foot complication that can lead to ulceration and potentially amputation. (High risk foot project Report)*

Enablers and barriers to change for each case were discussed by interviewees. Enablers were predominantly involving the right people who had the authority to introduce the change and ensuring processes were introduced within the ward environment. Whereas the barriers stated by interviewees included lack of communication, cost or time.

*This process works because dieticians manage [it]. (C3)*

*I think the fact that we set up guidelines so well at the beginning and took the surgeons on that journey. (C2)*

*… making sure there’s a sustainable pathway for funding as there’s a bit of a cost to the training … (M1)*.

*but it was always an additional task on top of an already busy workload (M3)*

In all cases, the practice change has continued since its introduction with the assumption the change will remain ongoing. The significance and extent of change was discussed by interviewees and ranged from localised ward change to an alteration on an international product disclose statement. Some of the benefits would be felt by patients whereas others benefitted the health service.

*For patients*,* care can be managed locally and therefore there is a reduced burden of travel … (M4)*.

*For the surgeons it meant they were free to do urgent clinical surgical reviews. (C2)*

*Opportunities exist to expand this MoC [model of care] to all rural health facilities that have an AHA/CA/HW [Allied Health Assistant/Clinical Assistant/Health Worker] and access to a Podiatrist for management of high-risk patients (Report)*.

*Baxter professional guidelines include the information about use in hyperbaric chambers: The Infusor and the Intermate may also be used inside hyperbaric chambers. As long as the patient is exposed to the same changes in pressure as the Infusor or Intermate device there is no hindrance to the infusion. (Baxter PDS)*

### Cross-case analysis

Cross-case analysis identified many similarities and some differences (Table 1). Overall, more similarities were noted among the three research projects, particularly regarding the rationale and instigator of practice change. All cases commenced with the intention to improve service delivery.

**Table 1:** Between case analysis categorised by themes which lists the similarities and differences across the cases

Category	Similarities	Differences
Practice change	All cases introduced system or process change to improve service delivery.	Pharm trialed on volunteers not patients first, others trialed on patients
	Expanded scope for AH clinicians in 3 cases (Pharm, Pod, OccThy)	1 case within scope of practice (Diet)
Rationale for practice change	3 cases designed to improve or ensure recovery (Pharm, OccThy, Diet),	One case designed to maintain health by reducing risk of foot ulcers (Pod)
	Bottom-up approach: AHP identified clinical problem then sought the funding (Diet, Pharm, OccThy)	Top-down: funding received prior to project plan (Pod)
Instigator of change	Research literate clinician concerned with current practice (Pharm, OccThy, Diet)	With funding secured, local audit showed high amputation rate was a priority area (Pod)
		Staff not involved in the project were unaware it was a locally driven practice change (Diet)
Enablers/Barriers of change	Inclusion of stakeholders who identified the problem and instigated the change (All cases)	Specific barriers reported were contextual or discipline specific (e.g. AH clinicians could identify need and introduce change as they are close to practice)
Sustainability of change	All cases have sustained the practice change. Although some of the changes have been modified.	Reach of change ranged from local health service (Diet, Pod) to other HHSs (Occ Thy) to international industry change (Pharm)
	All cases had some sort of documentation to sustain the change (e.g. work practice instructions)	

*Pod *Podiatry, *Diet *Dietetics, *Pharm *Pharmacy, *Occ Thy *Occupational Therapy

## Discussion

This study used a multiple case study design to evaluate Evidence Briefs as a communication tool for practice change. Causal effect demonstrated by case studies is gaining traction in some quarters [26] so this study used the design to link the allied health-led research to the practice change described. Evidence from this evaluation shows the practice change described by the Evidence Briefs occurred at THHS and was still in place at the time of evaluation. However, the Evidence Brief’s effectiveness as a communication tool beyond the stakeholders conducting the research or implementing the change cannot be determined. Further evaluation is needed to identify its effectiveness as a communication tool for local hospital management.

A notable finding in this study is the short time lag between commencing the research and its adoption into clinical practice, compared to the reported research implementation timeframes of up to 17 years [27]. The time taken from approval (via ethics or quality improvement registration) to its introduction on the respective ward/area was less than two years. A lack of agreement on starting points, types of measures and things to measure has been demonstrated when measuring time lags in medical research [27]. In contrast, the allied health research evaluated in the current study used the same start and end point across the cases, was clinician-led and conducted in the environment into which it was translated, overcoming some of the complexities described in other studies [28]. This suggests research literate clinicians, conducting research within healthcare organisations are best placed to identify clinical issues, obtain the evidence to address the issue and to implement the change in practice required to fix the problem.

Using a short narrative to describe research impact is not new. The Research Excellence Framework in the United Kingdom used impact case studies in 2014 [29] and 2021 [30] to provide accountability for the investment in research and to produce evidence of the benefits of this investment. Also, some impact frameworks suggest the inclusion of a plainly written narrative as a component of impact measurement [12, 31]. The Evidence Briefs developed and used at THHS to describe the translation of research or quality improvement into clinical practice are currently only used to communicate practice change. However, accumulation of Evidence Briefs over time suggests they may be able to be used as a measure of change where each Evidence Brief represents a unit of change. For example, the number of Evidence Briefs produced in any given year could be used as one measure of research impact.

The Evidence Brief was nominated as a data collection tool for the THHS Research Strategy in 2023. A trial of collecting Evidence Briefs on quality improvement projects across all clinical streams at THHS in 2024 has resulted in a report summarising 17 completed Evidence Briefs for submission to management. The report suggests longer term impacts such as improved access and efficiency. (unpublished data) Future research efforts by this team will continue investigating how the Evidence Brief is used in other healthcare organisations with implementation across northern Queensland already commenced. Additional research will be needed to collect feedback from management to further refine the Evidence Brief and design a pathway for its communication.

There were numerous similarities across the case studies. For example, the incentive or rationale for the intended change in most EBs was that a clinician identified a clinical problem to be solved. An impact review of a Western Australian research translation funding program suggested a bottom-up approach to translation-triggered positive outcomes across the impact domains of advancing knowledge, collaboration, capacity building and changes in policy and practice [32]. The incentive to conduct the study varied across the cases, but the rationale for doing all projects was to improve service delivery. Another similarity across the cases was stakeholders conducting the research were also the ones introducing the clinical practice change.

This study investigated the translation of research findings into clinical practice. However, longer-term impacts were also evident. For example, three of the cases produced peer reviewed publications demonstrating an increase in research capacity. The case describing the occupational therapy-led paediatric burns rehabilitation has shown a sustained improvement in access to care for rural and remote patients [33] This evidence of the benefits of allied health research suggests the repository of Evidence Briefs could be evaluated against longer term impacts in future studies.

## Conclusion

This evaluation showed the Evidence Brief describes clinical practice change as a result of allied health research or quality improvement. Further evaluation is required to demonstrate the Evidence Brief’s effectiveness as a communication tool for informing healthcare management about the practice change. Potentially, each Evidence Brief could be considered a unit of change so that over time, accumulated Evidence Briefs may be used to measure clinical practice change resulting from research. Future research will focus on using Evidence Briefs more broadly across other disciplines within healthcare organisations as a communication tool and the potential to use them as a starting point for future impact assessments.

## Supplementary Information

Supplementary Material 1.

Supplementary Material 2.

Supplementary Material 3.

## Data Availability

The data that support the findings of this study are available from the authors upon reasonable request.
