# Supplementary material for: Development and evaluation of a tool (named Evidence Brief) to communicate allied health research translation
Source: BMC Health Serv Res. 2025 Oct 21;25:1385. doi: 10.1186/s12913-025-13421-1 (PMC12542262; doi:10.1186/s12913-025-13421-1)
Supplement: Supplementary file 1 — Supplementary Material 1. [file 12913_2025_13421_MOESM1_ESM.docx]

| Reporting item | Page number on which item is reported | Page number of justification for not reporting |
| --- | --- | --- |
| Describing the design |  |  |
| 1. Define the research as a case study | P5, Lines 103-4 |  |
| 1. State the broad aims of the study | P5-6, lines 114-7 |  |
| 1. State the research question | P5, lines 98-100 |  |
| 1. Identify the specific case(s) and justify the selection | P5, lines 104-9 |  |
| Describing the data collection |  |  |
| 1. Describe how the data were collected | P7, lines 149-155 |  |
| 1. Describe the sources of evidence used |  |  |
| 1. Describe any ethical considerations and obtainment of relevant approvals, access and permissions | P1, lines 21-5 |  |
| Describing the data analysis |  |  |
| 1. Describe the data analysis methods | P7, lines 160-172 |  |
| Interpreting the results |  |  |
| 1. Describe any inherent shortcomings in the design and analysis and how these might have influenced the findings | P15, lines 328-333 |  |
| 1. Consider the appropriateness of methods used for the question and subject matter and why it was that qualitative methods were appropriate | P5, lines 103-112 |  |
| 1. Discuss the data analysis | P13, lines 271-326 |  |
| 1. Ensure that the assertions are sound, neither over- nor under-interpreting the data | P15, lines 330-333 |  |
| 1. State any caveats about the study | P15, lines 328-330 |  |
