# Supplementary material for: Development and evaluation of a tool (named Evidence Brief) to communicate allied health research translation
Source: BMC Health Serv Res. 2025 Oct 21;25:1385. doi: 10.1186/s12913-025-13421-1 (PMC12542262; doi:10.1186/s12913-025-13421-1)
Supplement: Supplementary file 2 — Supplementary Material 2. [file 12913_2025_13421_MOESM2_ESM.docx]

Interview guide

| **Question** | **Prompts** |
| --- | --- |
| Where do you work |  |
| What is your role in dept |  |
| Are you aware of any (clinical) practice change in your department? Over the last X period... | If yes, ask the next question  If unaware, describe the research |
| Can you describe that change for me? | Who decided to change?  How/when did the change occur? Why did the change occur? - this might elicit its connection to research or if that is recognised  What steps happened to make the change? |
| Are you still using the new protocol/procedure? |  |
| For clinicians:  What do you think was the Most significant thing about the change | For whom is it significant (eg patient, department, health service etc)  Reach of the significant change |
| Is there a department procedure/protocol/workplace guideline/instructions written | Can we get a copy? |
| For managers:  Can you discuss the barriers and enablers for change as a result of a research project in your department? |  |
