# Supplementary material for: Development and evaluation of a tool (named Evidence Brief) to communicate allied health research translation
Source: BMC Health Serv Res. 2025 Oct 21;25:1385. doi: 10.1186/s12913-025-13421-1 (PMC12542262; doi:10.1186/s12913-025-13421-1)
Supplement: Supplementary file 3 — Supplementary Material 3. [file 12913_2025_13421_MOESM3_ESM.docx]

| Title | **Dietetic Assessment of all patients on fluid diets for 3 days** |
| --- | --- |
| Authors | **Sarah Deacon**, Dietician, Townsville Hospital and Health Service  **Natalie Moran**, Dietitian, Townsville Hospital and Health Service  **Bonney Laskey-Gilboy**, Dietitian, Townsville Hospital and Health Service  **Maree De Jong**, Dietician, Townsville Hospital and Health Service  **Shonnel Rothery**, Dietician, Townsville Hospital and Health Service  **Kristina Ahnon**, Dietician, Townsville Hospital and Health Service  **Melissa Whiting**, Dietician, Townsville Hospital and Health Service  **Theophilus I. Emeto**, Public health and Tropical Medicine, James Cook University  **Dr Tilley Pain**, Principal Research Fellow, Townsville Hospital and Health Service |
| THHS Strategic Pillars | - Provide high quality, person-centred care for northern Queensland. |
| National Accreditation Standards | - Standard 5: Comprehensive care. - Standard 8: Recognising and responding to acute deterioration. |
| Practice Issue | Prolonged use of fluid diets increases malnutrition risk in the hospital population. Many patients are prescribed fluid diets; often following gastrointestinal surgery, during acute gastrointestinal illness, or in patients who are unable to chew or swallow solid foods. Both clear fluid and free fluid diets in general are inadequate in all micronutrients and macronutrients. Therefore, they are recommended for use for no longer than 3 days. At TTH, patients can remain on fluid diets greater than 3 days without receiving any nutrition support from a dietitian. |
| Evidence | A quasi-experimental study of 57 patients receiving fluid diets was conducted at TTH. Patients were nominated as intervention group if they were receiving dietetic intervention and control group received the standard order of fluid pertinent for their diet code. Patients in the intervention group were assessed by a dietitian and prescribed extra fluid items or supplement drinks, dietetic advice, education, or alternative feeding (i.e. enteral or parenteral nutrition) as per clinical dietetic practice. The fluid consumption of all study patients was observed on the ward over 24 hours, using the validated Comstock 6-point visual estimation scale. Total energy and protein requirements were calculated for each patient using the Nutrition Education Materials Online equations for non-hypermetabolic patients based on the patients’ current or adjusted ideal body weight. Actual patient intake was calculated from collected data. A between group comparison was performed on the control and intervention groups. Patients receiving dietetic intervention received 75.9% of their energy requirements compared to control group with 18.1% based on median intakes (p<0.001) and 76.0% of their protein requirements compared to control group with13.8% based on median intakes (p<0.001). These results were consistent across age, BMI and fluid diet type. Therefore, dietetic intervention allowed up to 80% more energy and 95% more protein consumption for patients on fluid diets. |
| Practice change | The significance of these differences which were consistent across age, BMI and fluid diet type, has resulted in a change of clinical practice at The Townsville Hospital. As of February 2016, all patients on fluid diets for 3 days or longer are blanket referred for dietetic intervention to allow timely nutrition support. |
| Citation | Deacon S, Moran N, Laskey-Gilboy B, De Jonge M, Rothery S, Ahnon K, Whiting M, Emeto TI, Pain T. Dietetic intervention for inpatients on fluid-only diets helps to achieve nutritional requirements. Nutr Diet. 2018 Feb;75(1):17-23. doi: 10.1111/1747-0080.12357. Epub 2017 Jun 14. PMID: 29411492. |

Attachment 1

| Title | **Use of elastomeric pumps during hyperbaric treatment** |
| --- | --- |
| Authors | **Stephen Perks,** Senior Pharmacist, Townsville Hospital and Health Service  **Denise F Blake**, Emergency Physician, Townsville Hospital and Health Service  **Derelle A Young,** Registered Nurse Hyperbaric Unit, Townsville HHS  **John Hardman,** Registered Nurse Hyperbaric Unit, Townsville HHS  **Lawrence H Brown**, Adjunct Associate Professor,  Mt Isa Centre for Rural & Remote Health, James Cook University  **Iestyn Lewis**, Emergency Physician, Royal Hobart Hospital  **Tilley Pain,** Principal Research Fellow, Townsville Hospital and Health Service |
| THHS Strategic Pillars | - Provide high quality, person-centred care for northern Queensland. - Work collaboratively, embrace innovation and continuously improve. |
| National Accreditation Standards | - Standard 3: Preventing and controlling healthcare-associated infection. - Standard 4: Medication safety. |
| Practice Issue | Some patients have indications for continuous antibiotic infusion and concurrent hyperbaric treatment. These indications include diabetic and venous foot ulcers, myonecrosis, necrotizing skin infections and refractory osteomyelitis. Electronic infusion pumps have been demonstrated to fail under hyperbaric conditions. Therefore, usual practice is for the infusion devise to be unhooked when the patient receives hyperbaric treatment. The practice of removing the infusion devise has been extended to elastomeric pumps. However, there is no evidence demonstrating any effect of hyperbaric treatment on flow rate of elastomeric pumps. The issue is that removing the infusion device during hyperbaric treatment means these patients receive a reduced dose of antibiotics during that 24-hour period and extra manipulations of PICC lines increase risk of infection. |
| Evidence | An evaluation of the flow rate of the LV10 Baxter infusion pump under three hyperbaric conditions, and normobaric conditions was performed. The elastomeric pumps were secured to participants in a manner identical to a typical patient except the antibiotic solution was diverted to a container. Pumps and tubing were weighed before and after the test period as a proxy measure for flow. Flow rate was determined at sea level and three different commonly used hyperbaric treatment pressures and two different time periods. Results from the study showed mean flow rates in ml/hr (SD) were: 10.4 (0.5), 10.7 (0.4), 10.5 (0.5), 9.5 (0.4) from 0-2 hours and 10.4 (0.8), 9.4 (0.5), 10.4 (1.0), 10.5 (1.2) at 19-21 hours for the 101 kPa, 140 kPa, 180 kPa and normobaric conditions groups respectively. Two-factor ANOVA found no significant associations between flow rate and pressure (F=0.18, p=0.671) or time period (F=0.061, p=0.611). |
| Practice change | Baxter elastomeric LV 10 pump flow rates are not affected by commonly used hyperbaric treatment pressures and therefore, are no longer disconnected from patients on continuous 24-hour antibiotic infusion who undergo concurrent hyperbaric treatment at The Townsville Hospital. No official protocol was written up. Staff within the department agreed to the change of practice of not disconnecting the elastomeric pumps based on the publication of results. This means that patients can now receive their full dose of antibiotic therapy whilst getting concomitant hyperbaric therapy. Rather than missing out on 10-15% of their daily dose due to the infusors being disconnected for the duration of hyperbaric therapy. |
| Citation | Perks S, Blake DF, Young DA, Hardman J, Brown LH, Lewis I, Pain T. An assessment of the performance of the Baxter elastomeric (LV10) Infusor^TM^ pump under hyperbaric conditions. *Diving and Hyperbaric Medicine* 2017;47(1):33-37.  DOI:10.28920/dhm47.1.33-37 |

Attachment 2

| Title | **Evaluation of an OT-led paediatric burns**  **telehealth review clinic** |
| --- | --- |
| Authors | **Debra Phillips,** Senior Occupational Therapist, Townsville University Hospital  **Tilley Pain**, Principal Research Fellow, Allied Health, Townsville University Hospital  **Lauren Matheson,** Occupational Therapist, Townsville University Hospital  **Gail Kingston,** Assistant Director Occupational Therapy, Townsville University Hospital |
| THHS Strategic Pillars | - Provide high quality, person-centred care for northern Queensland. - Work collaboratively, embrace innovation and continuously improve. |
| National Accreditation Standards | - Standard 5: Comprehensive care. |
| Practice Issue | Burns are a common injury in children. Deep-partial or full thickness burns often require hospitalisation and surgical intervention followed by complicated post-surgical rehabilitation to achieve optimal functional performance, minimal scarring and full range of movement.^1^ Optimal outcomes following severe burns are best achieved through specialist occupational therapy (OT) intervention.^2^ OT plays a major role in the management of hypertrophic scarring, reduced joint range of motion, pain and functional ability following burn injuries.^2^ Rural and remote children are more likely to have increased complications following burn injury because specialist OT burn services are located in tertiary referral hospitals and are rarely available in rural and remote areas.^3,4^ This rural and remote inequity was the impetus to develop a new model of care. |
| Evidence | The OT Led Paediatric Burn Telehealth Clinic increased the frequency of clinical reviews, saved patient travel time, demonstrated family satisfaction, and reduced demand on Paediatric Surgeon appointments in paediatric surgical outpatient clinics.  Family and clinician perspectives obtained from this study confirm the benefits of a telehealth service for rural and remote children post burn injury. The research study also provides feedback regarding opportunities for service enhancement. It demonstrates that this expanded scope allied health telehealth model provides quality patient centred care and expert clinical advice within local communities and builds the skill and capacity of local clinicians. This telehealth model can be translated to other areas of health care and clinical subspecialties across Australia. |
| Practice change | The OT-Led Paediatric Burn Telehealth Clinic is an ongoing model of service delivery offered to patients and their families. The model includes an expanded scope role for the OT, freeing up appointments for paediatric surgeons to see patients on their waiting lists. To date the model has successfully increased the frequency of follow-up appointments for rural and remote paediatric patients.  Service enhancements that were indicated in the research such as including rural clinicians in the telehealth review and delegating clinical tasks to an AHA have been implemented where practical. Strategies to promote sustainability of this service delivery model are being explored. |
| Citation | Phillips D, Matheson L, Pain T, Kingston GA. Development of an occupational-therapy-led paediatric burn telehealth review clinic. Rural Remote Health. 2021 Aug;21(3):6223. doi: 10.22605/RRH6223. Epub 2021 Aug 16. PMID: 34392690 |

Attachment 3

Format based on the Australian Healthcare and Hospitals Association’s (<https://ahha.asn.au/policy-briefs/>)

Tilley Pain, Townsville HHS (presented at HSR Conference 2017, Surfers Paradise, QLD)

Townsville HHS Research Strategy (<https://www.townsville.health.qld.gov.au/research/for-researchers/stratefy-and-reports>)

National Safety and Quality Health Service Standards (<https://www.safetyandquality.gov.au/nsqhs-standards>)
